# Combining Text Mining of Long Constructed Responses and Item-Based Measures: A Hybrid Test Design to Screen for Posttraumatic Stress Disorder (PTSD)

**DOI:** 10.3389/fpsyg.2019.02358

**Published:** 2019-10-22

**Authors:** Qiwei He, Bernard P. Veldkamp, Cees A. W. Glas, Stéphanie M. van den Berg

**Affiliations:** ^1^Educational Testing Service, Princeton, NJ, United States; ^2^Department of Research Methodology, Measurement and Data Analysis, Faculty of Behavioural, Management and Social Sciences, University of Twente, Enschede, Netherlands

**Keywords:** posttraumatic stress disorder, text mining, item response theory, Bayesian framework, self-narratives

## Abstract

This article introduces a new hybrid intake procedure developed for posttraumatic stress disorder (PTSD) screening, which combines an automated textual assessment of respondents’ self-narratives and item-based measures that are administered consequently. Text mining technique and item response modeling were used to analyze long constructed response (i.e., self-narratives) and responses to standardized questionnaires (i.e., multiple choices), respectively. The whole procedure is combined in a Bayesian framework where the textual assessment functions as prior information for the estimation of the PTSD latent trait. The purpose of this study is twofold: first, to investigate whether the combination model of textual analysis and item-based scaling could enhance the classification accuracy of PTSD, and second, to examine whether the standard error of estimates could be reduced through the use of the narrative as a sort of routing test. With the sample at hand, the combination model resulted in a reduction in the misclassification rate, as well as a decrease of standard error of latent trait estimation. These findings highlight the benefits of combining textual assessment and item-based measures in a psychiatric screening process. We conclude that the hybrid test design is a promising approach to increase test efficiency and is expected to be applicable in a broader scope of educational and psychological measurement in the future.

## Introduction

Epidemiological research on mental illnesses such as posttraumatic stress disorder (PTSD) requires efficient methods to identify cases in large population-based samples (Shrout and Yager, 1989) because the diagnosis of the disorder is difficult to make and can involve expensive testing. A two-phase design can help on both accounts. The first phase involves a screening measure, meaning a more detailed diagnostic procedure needs to be administered solely to a selected subsample (Diamond and Lilienfeld, 1962; Shrout et al., 1986).

Item-based self-report instruments are often considered efficient for PTSD screening, as they usually require short administration time and do not require the presence of a clinician (Wohlfarth et al., 2003). Questionnaires such as the Trauma Assessment of Adults (Gray et al., 2009), the Brief Trauma Questionnaire (Schnurr et al., 2002), the Life Events Checklist (Gray et al., 2004), and the Trauma Life Events Questionnaire (Kubany et al., 2000) all have psychometric support for evaluating exposure to potentiality traumatic events. In addition to trauma exposure screeners, abbreviated PTSD symptom screeners are frequently used to determine the need for more in-depth clinical interviews (Lancaster et al., 2016). These include the Primary Care PTSD Screen (PC-PTSD; Prins and Ouimette, 2004), the Short Form of the PTSD Checklist-Civilian Version (Lang and Stein, 2005), the Trauma Screening Questionnaire (TSQ; Brewin et al., 2000), and the Short Post-Traumatic Stress Disorder Rating Interview (SPRINT; Connor and Davidson, 2001). These instruments ideally contain the minimal number of items necessary for accurate case identification, have simple decision rules to determine who passes and fails the screening, and are applicable to populations with varying prevalence of PTSD and experiencing different traumas (see more in reviews by Brewin, 2005; Lancaster et al., 2016).

As an alternative to such questionnaire-based screening, He et al. (2012) developed a computerized textual assessment system using text mining techniques, which was proved to be effective in analyzing open-ended writings regarding participants’ trauma history and physical symptoms. The main idea was to analyze the respondents’ textual input – the self-narratives describing traumatic experiences and impacts on their personal life to predict the risks of developing PTSD. In their study, the textual screening procedure resulted in a good agreement (82%) compared with a clinical structured interview in identifying the presence and absence of PTSD and yielded a higher sensitivity and positive prediction power than an itemized screening instrument.

With a growing body of research in learning patterns of language usage in psychiatric patients, textual input became recognized as an important additional source in the prediction of mental health (Pennebaker et al., 2003). For instance, Pennebaker (2001) found that linguistic markers, such as the use of negative-emotion words, cognition words, and insight words, predicted the future mental health of college students who wrote about traumatic events. Alvarez-Conrad et al. (2001) defined the presence of words relating to death and dying as an indicator of treatment-resistant PTSD. Consequently, the analysis of respondents’ textual input and linguistic elements might provide crucial information for understanding cognitive mechanisms associated with trauma and hold valuable potential to screen for and predict PTSD symptoms and subtypes. Properly developed technologies such as text mining are expected to help individuals to self-test and public health organizations to screen for possible mental health conditions and prompt further evaluation when warranted, potentially preventing disorders from becoming chronic, debilitating, and difficult to treat (Todorov et al., 2018).

The focus of this study is to assess to what extent text mining techniques can be applied in the PTSD screening phase and to establish the extent to which they result in better estimates and better prediction of true diagnosis compared to the use of a questionnaire alone. Specifically, we propose a two-stage hybrid test design using a Bayesian approach to combine text mining and item response modeling in one systematic framework, where an automated score based on textual analysis serves as input for a prior distribution of a latent trait associated with PTSD that is measured by a number of questionnaire items using an item response theory (IRT) model (Rasch, 1960; Lord, 1980). Bayesian methods are especially useful for the estimation of a hierarchical structure (refer to Mislevy, 1986; Zwinderman, 1991), which allows extra prior information to be added into the measurement with the aim to increase prediction accuracy. Models developed in the Bayesian framework have been applied broadly in psychological and educational assessments. For instance, Matteucci and Veldkamp (2013) integrated students’ background variables, such as scores obtained by the examinees from other tests, socioeconomic variables, and demographic variables as prior information to improve the accuracy of students’ ability estimates (van den Berg et al., 2013) combined self-report and clinical interview data in a Bayesian approach to increase measurement precision in identifying schizotypal symptoms. However, the inclusion of textual assessments as prior information has been rarely described in the literature.

The purpose of this study is twofold: first, to investigate whether the combination model of textual analysis and item-based scaling can enhance the classification accuracy of PTSD, and second, to examine whether the standard error of estimates could be reduced through use of narrative as a kind of routing test. To examine the performance of our proposed method, we conducted a study to compare the estimates for a latent trait associated with PTSD with and without the use of a text mining score by means of three approaches: (1) an IRT-based test only, (2) textual analysis only, and (3) a combination of textual analysis and IRT-based itemized test including using the whole range of IRT-based items at one time and adding items adaptively starting from the one with the highest information, which is similar to the item selection procedure used in computerized adaptive testing (van der Linden and Glas, 2000).

## Materials and Methods

### Sample and Instrument

Data used in the current study were collected from 105 trauma survivors via an online survey embedded in an open forum that is dedicated to people with mental health issues. Before administering items from the survey, all the participants were asked to report whether they had been diagnosed as PTSD or non-PTSD by psychiatrists via structured interviews with standardized instruments. Cases with missing diagnoses were discarded in the present study. Participants were also informed that the objective of the research was to develop a more flexible intake procedure for PTSD diagnosis and were requested to give responses to all the questions following the instructions.

The online survey consisted of two parts: self-narrative writing and administration of dichotomous questions regarding PTSD symptoms. In the writing section, respondents were asked to write about their traumatic events and briefly describe the symptoms related to these experiences. Text length was recommended to be over 150 words, which was found as the average length of self-narratives input by PTSD patients in a previous study (He et al., 2012). In the item-based section, respondents were required to give compulsory answers to 21 items that were employed exactly the same in the National Comorbidity Study-Replication (NCS-R; Kessler et al., 2004) PTSD screening section. The NCS-R, conducted between February 2001 and April 2003 in the United States, is a nationally representative community household survey of the prevalence and correlates of mental disorders. These 21 dichotomous items (i.e., “yes” = 1, “no” = 0) one-to-one correspond to the PTSD symptoms that were defined in Diagnostic and Statistical Manual of Mental Disorders Fourth Version (DSM-IV; American Psychiatric Association, 2000). The first two columns in Table 1 show the PTSD diagnostic criteria in the DSM-IV and their corresponding items that were used in the NCS-R as well as in this study.

**TABLE 1:** Item Parameters of 21 Questions Related to PTSD in NCS-R (calibrated with *n* = 880).

**Item**	**Question in NCS-R**	**α**	**SE (α)**	**β**	**SE (β)**	***r***
A2	Did you feel terrified or very frightened, helpless, shocked or horrified, numb at the time?	1.19	0.41	–4.45	0.48	0.19
B1	Did you ever have repeated unwanted memories of the event, that is, you kept remembering it even when you didn’t want to?	1.82	0.20	–1.74	0.15	0.58
B2	Did you ever have repeated unpleasant dreams about the event?	1.24	0.14	–0.49	0.10	0.51
B3	Did you have flashbacks, that is, suddenly act or feel as if the event were happening over again?	1.41	0.15	–0.22	0.10	0.54
B4	Did you get very upset when you were reminded of the event?	1.64	0.18	–1.18	0.12	0.56
B5	When you were reminded of the event, did you ever have physical reactions like sweating, your heart racing, or feeling shaky?	1.68	0.17	–0.34	0.11	0.58
C1	After the event, did you try not to think about it?	0.95	0.12	–1.31	0.11	0.42
C2	After the event, did you purposely stay away from places, people or activities that reminded you of it?	1.34	0.14	–0.45	0.10	0.52
C3	After the event, were you ever unable to remember some important parts of what happened?	0.83	0.10	0.58	0.08	0.39
C4	After the event, did you lose interest in doing things you used to enjoy?	1.53	0.15	–0.39	0.10	0.53
C5	After the event, did you feel emotionally distant or cut-off from other people?	1.55	0.16	–0.88	0.11	0.53
C6	After the event, did you have trouble feeling normal feelings like love, happiness, or warmth toward other people?	1.86	0.18	–0.55	0.12	0.58
C7	After the event, did you feel you had no reason to plan for the future because you thought it would be cut short?	1.45	0.15	1.22	0.12	0.47
D1	During the time this event affected you most, did you have trouble falling or staying asleep?	1.14	0.18	–1.53	0.12	0.39
D2	During the time this event affected you most, were you more irritable or short-tempered than you usually are?	1.11	0.14	–0.16	0.09	0.46
D3	During the time this event affected you most, did you have more trouble concentrating or keeping your mind on what you were doing?	1.47	0.19	–1.10	0.11	0.48
D4	During the time this event affected you most, were you much more alert or watchful, even when there was no real need to be?	0.96	0.16	–0.85	0.10	0.39
D5	During the time this event affected you most, were you more jumpy or easily startled by ordinary noises?	1.28	0.17	–0.55	0.10	0.49
E1	Was any of these reactions continue to have at least 1 month?	0.78	0.30	–3.30	0.21	0.21
F1	Did these reactions cause distress to you?	1.55	0.26	–2.15	0.17	0.38
F2	Did these reactions disrupt or interfere with your normal, daily life?	1.02	0.16	–0.88	0.11	0.40

*The item parameters were estimated from unidimensional 2PL model on a sample of 880 respondents in the NCS-R. SE indicates the standard error of item parameter estimation. *r* indicates validity coefficients that are calculated as the correlation of total score with each criterion item.*

Six of the 105 participants were excluded: Two reported they had never experienced traumatic events that were listed in the NCS-R, and four gave responses only to the item section but missed the writing section. This resulted in a total of 99 participants for the final set, among whom 34 were diagnosed as PTSD and 65 as non-PTSD. The sample had an age range between 19 and 63, with a mean of 30.06 (*SD* = 11.30). The majority of participants were female (78.4%). Over 90% participants had a higher educational background (i.e., college/university or above). 52.6% participants were reported as single, 40.2% were married, and 6.2% were divorced.

### Procedure

To examine the performance of the hybrid test design, we estimated individuals’ PTSD latent traits via three approaches: (1) an IRT-based test only, (2) text classification of self-narratives, and (3) combining textual analysis and IRT in a Bayesian framework. There were two analytic paths involved in the third approach: In one path, we combined the textual analysis with the whole set of 21 IRT-based items at a single time. In the other, we combined the textual analysis and the IRT latent scale in an adaptive way, that is, we added the 21 items into the analysis one by one in descending order of item information available. We will illustrate each approach in detail in the following subsections. All analyses in the Bayesian framework were conducted using the software WinBUGS 1.4.3 (Lunn et al., 2000).

#### Approach 1: Using an IRT-Based Test Only

The IRT framework has been increasingly applied in psychiatric assessments in recent decades (e.g., van Groen et al., 2010; Weisscher et al., 2010; He et al., 2014b). In contrast to the classical sum score methods, IRT models (Rasch, 1960; Lord, 1980) provide improvement and flexibility by scaling the difficulty of items and the latent trait level of people on the same metric. Namely, the severity of prescribed symptoms and the latent degree of individuals’ mental illness are set on a common scale, and thus can be meaningfully compared.

In the first approach, we focused on applying an IRT model on responses to the 21 PTSD diagnostic items in the NCS-R without adding any prior information. We employed a set of fixed item parameters that were previously calibrated using a larger sample size of 880 respondents collected in the NCS-R (He et al., 2014b). Note, however, that these 880 respondents gave responses to the questionnaire only, without any input by way of self-narratives. Given the objective of this study – examining the role of textual information in latent trait estimation to screen for PTSD, we had to collect a new sample of 99 respondents in this study who gave responses to both textual self-narratives and an itemized questionnaire, thus making it possible to combine both structured and unstructured data analysis in one framework.

In He et al. (2014b), given that symptom domains defined by the DSM-IV were used to index a general level of PTSD severity, we first considered a unidimensional two-parameter logistic (2PL) model underlying responses to the 21 symptoms (i.e., all 21 items on a single dimension). Next, given that the major 17 symptoms (in criteria domains B, C, and D) are placed *a priori* into three separate criterion domains, we also considered a three-dimensional IRT model where each domain was associated with a separate dimension. In addition, a special version of the 2PL model – the Rasch model or one-parameter logistic (1PL) model (Rasch, 1960) where the item discrimination parameter is simply fixed as one – was also considered, since such a model is often used in clinical applications as well (e.g., Wong et al., 2007; Elhai et al., 2011).

In the unidimensional 2PL model, that is, the probability of a score in category “yes” (*X*_*ni*_ = 1) of item *i* is given by the item response function

(1) $$P\left(X_{n\,i}=1|\theta_{n}\right)=\frac{\exp\left[\alpha_{i}\,\left(\theta_{n}-\beta_{i}\right)\right]}{1+\exp\left[\alpha_{i}\,\left(\theta_{n}-\beta_{i}\right)\right]},$$

where θ_*n*_ is the latent PTSD level of person *n*, β_*i*_ is an item difficulty parameter representing the severity level of each diagnostic symptom, and α_*i*_ is an item discrimination parameter indicating the extent to which the item response is related to the latent θ-scale. Note that in the Rasch model, the discrimination parameter α_*i*_ is fixed as 1. In the multidimensional version of the 2PL model, the probability of a positive response depends on *M* latent variables, say θ_*n*1_,…,θ_*nm*_,…,θ_*nM*_. In the multidimensional case, in eq. 1, the product α_*i*_θ_*n*_ is replaced by $\sum_{m}{\text{α}}_{im}{\text{θ}}_{nm}$.

The dimensionality and model fit were examined using two steps: a likelihood ratio-statistic and an item-oriented Lagrange multiplier (LM) test. First, the likelihood-ratio test of the 2PL model against the Rasch model yielded a value of the test statistic χ^2^ = 78.53,*df* = 16,*p* < 0.001, while the multidimensional model against the unidimensional 2PL model yielded a value of χ^2^ = 37.41,*df* = 3,*p* < 0.001. It was concluded that the multidimensional model fit the data best, and the 2PL fit the data significantly better than the Rasch model. However, although using a more complex model generally results in better model fit, using a more parsimonious model might still lead to adequate data description.

To investigate this, a second approach was used. Under each model, item fit was evaluated using an LM item fit statistic (Glas, 1998, 1999). These statistics can be used to evaluate the fit of the expected item response function given by Formula (1) to the observed item responses. Item fit was tested with a significance level of 0.01. For the Rasch model, the test was significant for six items, while no tests were significant for either the 2PL model or the multidimensional model. Further, the LM test statistic is accompanied by an effect size that measures the difference in observed and expected average item responses. For the 2PL model and the multidimensional model, these differences had the same magnitude. Hence, although a multidimensional IRT model fit the data better than 2PL in terms of the likelihood ratio test, it was not clearly superior in item fit. Therefore, the simpler unidimensional 2PL model was preferred over the more complicated multidimensional one. Consequently, the item calibration in the NCS-R was undertaken with the unidimensional 2PL model by marginal maximum likelihood (Bock and Aitkin, 1981) on a sample of 880 respondents in He et al. (2014b).

Further, we calculated validity coefficients *r* to examine how strong each criterion weighed on the general trait of PTSD and check whether these external criteria could match the discrimination parameters derived from the 2PL that indicates the extent to which the item response was related to the latent θ-scale. The validity coefficient is a statistical index used to report evidence of validity for intended interpretations of test scores and defined as the magnitude of the correlation between test scores and a criterion variable. We calculated the validity coefficients as the correlations between the NCS-R test results and each criterion variable and reported the results in the last column in Table 1. The larger the validity coefficient, the more confidence we can have in predictions made from the PTSD test scores. As shown in Table 1, the discrimination parameters in the third column showed a high agreement with the validity coefficients in the last column: for instance, the highest discrimination parameter located in criterion C6, where the top validity coefficient 0.58 was also found in this item. Similar findings were also applied to the lowest values of these two variables such as in criterion E1 and C3. The evidence demonstrated that the item weighting from the 2PL could provide similar conclusions based on external criteria (i.e., validity coefficients) to get consistent results in identifying strong (weak) factors in the test.

To maintain consistency with the previous study (He et al., 2014b), we fixed the calibrated item parameters in the current study. The fixed parameters and their standard errors were reported in the third column to the sixth column in Table 1. As shown here, the discrimination parameters varied in the interval [0.78, 1.86], with a mean value around 1.32. The difficulty parameters were included in the range [−4.45, 1.22], with a mean of −0.99. The respondents’ latent traits were estimated by expected *a posteriori* (EAP) assuming a normal distribution.

#### Approach 2: Text Classification of Self-Narratives

Text classification is a special approach in the field of text mining, aiming to assign textual objects from a universe to two or more classes (Manning and Schütze, 1999). Supervised text classification generally involves two phases: a training phase and a testing phase. During the training phase, the most discriminative keywords to determine the presence or absence of PTSD are extracted and the relationship between the keywords and class labels is learned. The testing phase involves checking how well the trained classification model performs on a new dataset. In the testing procedure, each new input is scanned for the keywords that were extracted from training, and the most likely label for each new self-narrative is predicted. He et al. (2012) developed a supervised text classification model for PTSD screening. In this study, 300 self-narratives, consisting of 150 written by PTSD respondents and 150 written by non-PTSD respondents, were used to develop a screening system. In a follow-up study (He et al., 2017), four machine learning algorithms – including Decision Tree (DT), Naïve Bayes (NB), Support Vector Machine (SVM), and a self-developed alternative, the product score model (PSM) – were employed in conjunction with five data representations – unigrams, bigrams, trigrams, a combination of uni- and bigrams, and a mixture of n-grams. Unigram is the simplest and most commonly used data representation model where each word in a document collection acts as a distinct feature. N-gram considers the interaction effect of two, three, or more consecutive words (Manning and Schütze, 1999).

In He et al. (2017), it was found that narrative classification accuracy was maximized with the PSM in conjunction with unigrams. Although the addition of n-grams (i.e., bigrams and trigrams) has not significantly enhanced overall classification accuracy, it did help balance the performance metrics of text classification and improve the reliability of prediction. Furthermore, slight prevalence effects were found in the overall accuracy of all four machine learning algorithms; however, a substantial increase of positive prediction value (PPV) was noticed with the increase of prevalence of PTSD. When the prevalence of PTSD was low, the SVM and PSM had good sensitivity and high negative predictive power. This suggested that these two models could perform well in excluding the individuals identified as non-PTSD from the follow-up tests. Further, in a comparison with the mean performance of traditional screening measures reviewed by Brewin (2005), the SVM and PSM were shown to be more sensitive in detecting PTSD than the traditional screening measures, but their ability in detecting non-PTSD was a bit lower than the benchmark in clinical practice.

Because the PSM in conjunction with unigrams resulted in the highest agreement with the psychiatrists’ diagnoses in clinical practice in the previous study (He et al., 2017), we applied this approach in the present study. We used the top 1,000 unigrams that were identified as the most robust classifiers to distinguish PTSD from the non-PTSD in He et al. (2012, 2017). Among the 1,000 unigrams, in descending order of word frequency, the 10 unique words most used by the PTSD patients were “rape,” “flashback,” “fire,” “involve,” “avoid,” “incident,” “date,” “tower,” “men,” and “fault.” The words “test,” “hardly,” “tumor,” “tight,” “excite,” “evil,” “pleasure,” “vision,” “frantic,” and “funny” were found to be the top 10 in the non-PTSD corpus (He et al., 2012). Analogous to the results obtained by Orsillo et al. (2004) in the research regarding emotion expressions of PTSD patients, the words favored by PTSD patients had relatively stronger negative semantic tendency no matter the lexical form: adjective, noun, or verb (He et al., 2012).

A preprocessing routine was implemented to standardize the n-grams for textual analysis, which was consistent with the previous studies (He et al., 2012, 2017). This involved screening digital numbers, deducting non-informative “stop words”^1^ (e.g., “*I*,” “*to*”), common punctuation marks (e.g., “,” “*:*”) and frequently used abbreviations (e.g., “*isn’t*,” “I’m”), and “stemming” the rest of the words, using the Porter algorithm (Porter, 1980), to remove common morphological endings. For example, the terms “*nightmares*,” “*nightmaring*,” and “*nightmare*” were normalized in an identical stem “*nightmar*”^2^ by removing the suffixes and linguistic rule-based indicators (for more preprocessing rules refer to Manning and Schütze, 1999; He et al., 2012, 2017).

The PSM is an alternative machine learning algorithm to address the smoothing issue of NB using a form of Laplace’s law (Laplace, 1995). This model was validated in previous studies (He et al., 2012, 2017). Holding the similar independence assumption as the NB model, the PSM features assigning two weights for each keyword (in binary classification) to indicate how popular the keywords are in the corpora of self-narratives written by either PTSD patients (corpus^3^ *C*_*1*_) or non-PTSD patients (corpus *C*_*2*_). The name *product score* comes from a product operation to compute scores for each class, that is, *S*_*1*_ and *S*_*2*_, for each input text based on the term weights. To be consistent with the previous studies, we used the smoothing constant *a* = 0.5, which was added to the word frequency to account for words that did not occur in the training set but might occur in new texts (for more smoothing rules refer to Manning and Schütze, 1999; Jurafsky and Martin, 2009). The equation is,

(2) $$\left\{ \begin{array}{c} S_{1}=P\,\left(C_{1}\right)\cdot \prod_{w=1}^{k}\left[\left(u_{w}+a\right)/l\,e\,n\,\left(C_{1}\right)\right]\\ S_{2}=P\,\left(C_{2}\right)\cdot \prod_{w=1}^{k}\left[\left(v_{w}+a\right)/l\,e\,n\,\left(C_{2}\right)\right], \end{array}\right.\;$$

where *u*_*w*_ and *v*_*w*_ are the number of occurrences of keyword *w* in both corpora *C*_*1*_ (i.e., PTSD corpus) and *C*_*2*_ (i.e., non-PTSD corpus), respectively. *len*(*C*) is the corpus length, namely, the sum of the word occurrences in each corpus. *P*(*C*) is the prior probability of a certain class in the whole corpus collection. The classification rule is defined as:

(3) $$\mathrm{choose}\,\left\{ \begin{array}{c} C=1\;\mathrm{if}\,\log\left(S_{1}/S_{2}\right)>b\\ C=2\,\mathrm{else} \end{array}\right.,$$

where *b* is a constant set as zero in this study. The reason was that in the previous study (He et al., 2012) it was found during the PTSD textual screening procedure that the largest number of positive cases could be captured without unduly sacrificing specificity when the threshold was set at zero. The value of *log*⁡(*S*_1_/*S*_2_) was defined as the text score for each self-narrative (see also He and Veldkamp, 2012; He et al., 2012). For an easy comparison with the IRT scales, we standardized the text scores as *Z*∼*N*(0,1)^4^.

#### Approach 3: Combining Textual Analysis and IRT in a Bayesian Framework

Textual analysis and item response modeling were combined in a Bayesian framework, where the text score of each self-narrative obtained in approach 2 was used as prior information. The posterior distribution of the latent PTSD level is proportional to the product of the prior and the likelihood, that is,

(4) P⁢(θ|x,y)∝p⁢(x|θ,α,β)⁢g⁢(θ|y),

where *x* is the vector of responses to the questionnaire, *y* is the text score for each individual, *g*(θ|*y*)is the prior given the covariate of textual assessments, α and β are the fixed discrimination and difficulty parameters of items, *p*(*x*|θ,α,β) is the likelihood function of the IRT model. The relation between the PTSD latent trait θ of individual *n* and the text score *y*_*n*_ is given by the linear regression

(5) $${\text{θ}}_{\text{n}}=b_{0}+b_{1}y_{n}+{\text{ε}}_{n},$$

where *b*_*0*_ and *b*_*1*_ are the regression coefficients. The error terms are assumed to be independent and normally distributed as ε_*n*_∼*N*(0,σ^2^) with *n* = 1,…,*N* individuals. The assumption of a linear regression model is translated into a normal conditional distribution of θ_*n*_ given the text covariate as

(6) $$\theta_{n}|y_{n}∼N\,\left(b_{0}+b_{1}\,y_{n},\,\sigma^{2}\right)$$

Formula (6) represents an informative prior distribution of the PTSD latent trait. For each individual, the estimation of latent trait was performed by using 5,000 Markov chain Monte Carlo (MCMC) iterations with the burn-in of length of 1,000.

To determine whether the introduction of the prior distribution was effective, we compared the posterior distribution of θ_*n*_ in the combination model with the estimation from the IRT-based test only. Because the item parameters in the IRT model were fixed, the θ-estimates resulting from both of the IRT-based test and the combination model (use textual information as a prior) were on a common scale and thus could be compared.

Two investigations were conducted to analyze the efficiency of the combination model. The first was to combine the textual assessments with the full range of 21 items of the NCS-R questionnaire. The main purpose was to explore whether adding the text prior would significantly impact the accuracy of PTSD detection. The second investigation pursued the question of whether adding textual assessments to the questionnaire could result in a reduction of the number of items administered without sacrificing precision of the θ-estimates. Those items that provide peak information around the cutoff threshold are ideal for a shorter version of a mastery test (Thomas, 2011). Since the target of screening is to make classification decisions, a natural choice would be to maximize information at the chosen diagnostic cutoff (for more about item information refer to Lord, 1980). In the current study, we employed the same cutoff point at θ = −0.15 that was derived from He et al. (2014b) to distinguish PTSD and non-PTSD using a larger sample size of 880 respondents collected in the NCS-R. As mentioned above, this study shared the same questionnaire scale and item parameters as He et al. (2014b). This ensured the value of the cutoff point was comparable in these two studies. Further, the cutoff point derived based on a larger sample size was shown to be more reliable than a smaller sample size, so we kept the cutoff value consistent.

In He et al. (2014b), three approaches were used to set the standard (i.e., obtain a cutoff point on the latent scale) to distinguish PTSD and non-PTSD. The first approach entailed finding the midpoint between the medians of the two distributions (Cizek and Bunch, 2007). The second was the contrasting-groups method (Brandon, 2002), which uses logistic regression to determine the latent score point at which the probability of category membership is 50%. Setting the respondent status as a dichotomous variable coded 0 = non-PTSD and 1 = PTSD, we entered the latent scores of all the respondents into a general logistic regression equation; that is, *y*^∗^ = *a* + *bx*, where *y*^∗^ is the predicted value of the outcome variable (respondent status) for a respondent and *x* is the respondent’s observed score. Given *y*^∗^ = 0.5, the classification cutoff point for PTSD and non-PTSD groups could be obtained simply. The third approach used the Bayesian discrimination function, which minimizes expected risk. Using the zero-one loss function, the decision boundary becomes $g_{i}\,\left(x\right)=P\,\left(C_{i}|x\right)=\frac{P\,\left(C_{i}\right)\,p\,\left(x|C_{i}\right)}{p\,\left(x\right)}$, where *P*(*C*_*i*_) is the prior probability (i.e., the prevalence of PTSD or non-PTSD in the total sample); *p*(*x*|*C*_*i*_) represents the class likelihood (we assumed the latent trait scores have a normal distribution); and *p*(*x*) indicates the marginal probability of observation *x*. Given the assumption of normal distribution in both PTSD and non-PTSD groups, we could derive the cutoff point. Finally, we calculated the average of these three cutoff points based on the 21 items in the NCS-R and got −0.15 as the cutoff point on the latent scale.

**FIGURE 1:**
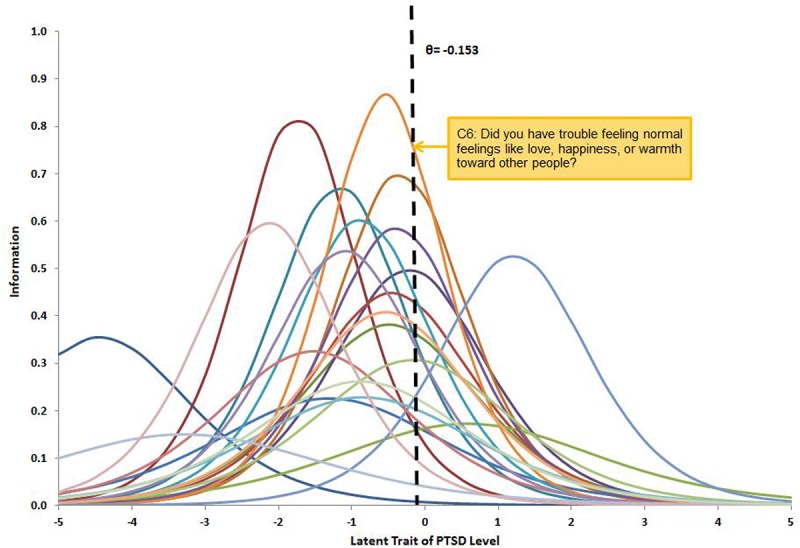
Item information for 21 items in NCS-R questionnaire corresponding to DSM-IV PTSD diagnosis criteria. The cutoff point was estimated at −0.15 on latent scale to distinguish PTSD and non-PTSD. Item C6 is the most informative item, having the highest intersection value with the cutoff line.

Consequently, in the present study, we calculated the item information for all the 21 items at this derived cutoff point and ranked the items in a descending order, namely, starting from the item with the highest information to the least information (see Figure 1). The items were ordered as following: C6, B5, C4, B3, C5, C2, D5, B2, B4, D3, D2, F2, C7, D4, D1, C1, B1, C3, F1, E1, A2. We started to examine the performance of a combination of the text prior and the most informative item – text prior with item C6 (i.e., “did you have trouble feeling normal feelings like love, happiness, or warmth toward other people?”) versus using item C6 alone. The second informative item (B5) was then added in for the comparison of the next pattern. The procedure continued until all the 21 items were included. Both test information and standard error of θ – estimates were calculated for each pattern (i.e., with and without text prior) with an increasing number of informative items. Since textual assessment was suggested as a sort of complementary information to predict people’s physical and mental health (e.g., Gottschalk and Gleser, 1969; Rosenberg and Tucker, 1979; Smyth, 1998; Franklin and Thompson, 2005), the test information was expected to increase, and the standard errors were expected to decrease when text priors were added.

The performance of the three approaches was compared on five metrics: accuracy, sensitivity, specificity, positive predictive value (PPV) and negative predictive value (NPV). The diagnoses made in the structured interviews by psychiatrists were used as the true standard in the comparison. Accuracy, the main metric used in classification, is the percentage of correctly defined individuals. Sensitivity and specificity are the proportion of actual positives and actual negatives that are correctly identified, respectively. These two indicators do not depend on the prevalence in the sample (i.e., proportion of “PTSD” and “non-PTSD” of the total), and hence are indicative of real-world performance. The predictive values, PPV and NPV, are estimators of the confidence in predicting correct classification, that is, the higher predictive values are, the more reliable the prediction is.

## Results

For the sample of 99 participants, the latent trait estimation via approach 1 resulted in a normal distribution of latent trait levels θ_*n*_, with a mean value of −0.39 and variance of 2.31. The standardized text scores obtained from approach 2 resulted in a range [−2.92, 4.22]. In approach 3, the latent linear regression model given by Formula (4) and (5) was estimated using the item responses and the textual covariates. The intercept and slope coefficients were obtained as −0.41 and 1.44, respectively. The error term in the prior information (textual covariates) had a normal distribution with a mean value of zero and variance of 3.57. Hence, the informative prior distribution of the PTSD latent trait was defined as θ_*n*_|*y*_*n*_∼*N*(−0.41 + 1.44*y*_*n*_,3.57).

The correlations among the estimations from the three approaches are presented in Table 2. It was noted that the correlation between the EAP of θ-estimates via approach 1 and the text scores estimated via approach 2 was 0.56, suggesting that there was a positive and moderate relation between the self-narrative writing and the responses to the itemized questionnaire in the structured interview. This result reiterated the findings in the earlier studies that the words and expressions were capable of predicting one’s mental health status.

**TABLE 2:** Correlations among estimates from three approaches: IRT, TX, and a combination of TX and IRT (21-item).

	**IRT**	**TX**	**TX and IRT (21-item)**
IRT	1.00		
TX	0.56	1.00	
TX and IRT (21-item)	0.99	0.62	1.00

*TX indicates the textual assessments. Correlation is significant at the 0.01 level (2-tailed).*

Table 3 shows the performance metrics of the three approaches. As we expected, the diagnostic accuracy rate was fairly high – 0.94 – when using the 21-item questionnaire by the IRT alone, and was improved to 0.97 with an addition of textual assessment. It suggested that 6 out of 99 respondents were misclassified using the IRT scale alone, while the misclassification rate decrease to 3 out of 99 respondents when adding the textual analysis as prior information. Using a 95% confidence interval, the paired sample *t*-test showed that the mean of latent trait estimation (*t* = 3.86, *df* = 98, *p* < 0.01) and standard deviation of latent trait distribution both significantly differ with and without text prior (*t* = 3.70, *df* = 98, *p* < 0.01). That is, the extra information gained from the textual analysis helped the latent trait locate closer to their true value, which helped decrease the misclassification rate by 50%. Given concerns on only using the keywords as predictors to make the classification, the accuracy rate (0.84) produced by the textual assessment was satisfactorily high, although it was a bit lower than the other two approaches. The sensitivity and NPV were perfect for all three approaches, implying that both the IRT and the textual assessments were sensitive for identifying PTSD patients. With the introduction of textual assessment, the specificity and PPV rose to 0.95 and 0.92, respectively. It suggested that the textual assessment played an effective role in detecting non-PTSD and strengthened the power in identifying PTSD in the population.

**TABLE 3:** Performance metrics compared among IRT, TX, and a combination of TX and IRT (21-item).

	**Accuracy**	**Sensitivity**	**Specificity**	**PPV**	**NPV**
IRT	0.94	1.00	0.92	0.87	1.00
TX	0.84	1.00	0.77	0.69	1.00
TX and IRT (21-item)	0.97	1.00	0.95	0.92	1.00

*TX indicates textual assessment. PPV and NPV represent the positive predictive value and negative predictive value, respectively.*

**FIGURE 2:**
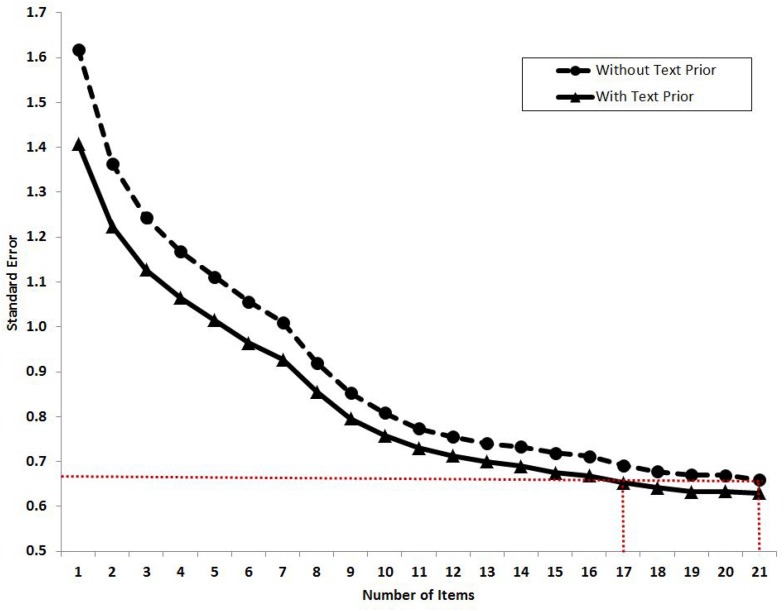
The relationship between standard error of the estimate of θ and the increasing number of items with or without using text priors. The red dotted line indicates the standard error when using 21 items without text priors. It crosses the solid standard error curve at 17 items, meaning that by using the text priors, the test length can be shortened by four items. The order of items is ranked by a descending order of item information with the cutoff point that was derived in Figure 1.

We further examined the relationship between the standard error of the estimate of θ and the number of items with the presence or absence of text prior. We added in items into the analysis one by one following an adaptive way with a descending order of the item information, which was derived at the cutoff point introduced in the Figure 1. As shown in Figure 2, the horizontal axis indicates the number of items in the IRT model and the vertical axis indicates the average standard error of the latent trait estimation. The curve of standard error without using the text prior (i.e., the dash line), that is, using the IRT model alone via approach 1, starts around 1.6 and drops gradually to 0.68 when all the 21 items are included. The curve of standard error using a text prior (i.e., the solid line) follows a similar pattern but stays on a lower level than the dash curve. It starts around 1.4 (when the first item with the highest information was included) and ends around 0.65 (when all the 21 items were included). Using a 95% confidence interval, the paired sample *t*-test showed that the standard error of estimation with text prior was significantly lower than that without text prior (*t* = 3.86, *df* = 98, *p* < 0.01) when including the whole range of 21 items. With the increasing number of items, the differences between these two curves decreased from 0.20 to 0.03. It suggested that the textual assessment did have an impact on the latent trait estimation, and the effect was more apparent when using fewer items. The red dotted line highlights the standard error when using 21 items without the text prior. It crosses the solid curve at 17 items, implying that with the introduction of the text prior, 17 items would be good enough to make the estimation as precisely as using the whole range of 21 items. That is, by using the text priors, the questionnaire length can be shortened by 4 items without sacrificing precision.

## Discussion

In this study, a new intake procedure for PTSD screening was developed that combined an automated textual assessment of patients’ self-narratives and an itemized questionnaire. To determine whether the introduction of text information is effective, we identified PTSD cases via three approaches: (1) we estimated PTSD latent trait by using IRT on a standardized questionnaire, (2) classified patients’ self-narratives into PTSD and non-PTSD groups by using a text mining technique, and (3) estimated the posterior distribution of PTSD latent trait by combining textual assessments and IRT in a Bayesian framework by both a linear and adaptive method. With the sample at hand, the results showed that the combination model enhanced the accuracy of PTSD detection from 0.94 to 0.97, reduced the standard error of latent trait estimation, and could shorten the questionnaire length by four items without sacrificing accuracy.

In the current study, the diagnostic accuracy was already high (0.94) when using the itemized questionnaire alone (approach 1). However, a structured interview that generally employs questionnaires is time consuming in daily practice. The computerized textual assessment proposed in this study is relatively easy to conduct via the internet. The highly satisfactory detection accuracy rate (0.84) is promising for real application. Note that the threshold in textual analysis could be adjusted according to the requirements of the practioner, for instance, using a relatively lower threshold to include the maximum number of PTSD potential patients for the second step in an itemized questionnaire, or increasing the threshold to a higher value in order to precisely detect PTSD patients by the textual assessment alone (He et al., 2012). Given concerns of the cost-effectiveness of the screening at an initial stage, it would be interesting to combine these two approaches in a two-phase framework to reduce clinical expense and improve the accuracy rate.

Further, according to the results in the previous study of He et al. (2012), the NPV of the textual assessments was satisfactorily high – 0.85 – when the text classification algorithm PSM was applied in conjunction with unigrams. It meant that the textual screening tool was helpful in excluding the non-PTSD respondents from the follow-up tests. For the 99 sample in the present study, taking the 85% confidence interval, 53 respondents could be excluded from the further tests.

It is also worthwhile to discuss the cost-effectiveness of the hybrid test design that combined the textual analysis and item-based test. The results showed that using textual information helped save follow-up items. However, weighing the benefits of the text prior, we would also take the amount of time it takes to write self-narratives into account. On the one hand, from respondents’ perspective, writing self-narratives provides flexibility to express the individual’s inner world and prevents being passively triggered by sensitive questions, even if the process might take longer than directly responding to the itemized questionnaire. On the other hand, from the practitioners’ perspective, the procedure for item development is often time consuming and involves multiple steps (e.g., data collection, data cleaning, field trial, item parameter calibration, and examination of reliability and validity of a scale). Comparatively, textual analysis could substantially shorten scale-development time and simplify the procedure once the model is successfully trained and refined with different textual contexts.

In addition, structured textual analysis that usually involves tight structures from existing software, such as Linguistic Inquiry and Word Count (LIWC; Pennebaker et al., 2001), is a good supplement to the text mining-based techniques. LIWC is a textual analysis software program that looks for words and counts them in categories relevant to psychology across multiple text files, for instance, essays, emails, blogs, novels, and so on. It has two central features – the processing component and dictionaries. During processing, the program goes through each file word by word. Each word in a given text file is compared with the dictionary file. A dictionary refers to the collection of words that define a particular category such as “family,” “positive emotion,” and “work.” In a pilot study based on 50 self-narratives, half written by a PTSD group and half by a non-PTSD group, it was found that the PTSD respondents used significantly more emotional words and expressions related to family. These results are interesting enough to be addressed in another paper in the future.

Some limitations in the present study also merit discussion. First, the sample size was rather small at only 99 participants. Second, it was notable that female respondents represented the majority (approximately 78%) in the sample, which was consistent with the proportion of females in the target sample of PTSD^5^ in the NCS-R. Further, evidence has shown that females are associated with a higher risk for PTSD (e.g., Lancaster et al., 2016). It would be interesting to examine whether the screening method (with text priors) plays an equal role in detecting PTSD in males and females, especially given concerns about the potential differences in their writing habits. Third, those in the sample had an unusually high level of education. This was probably caused largely by data collection being conducted on an internet platform. People with a higher educational background are possibly easier accessed via a web-based test than a less educated group (Naglieri et al., 2004). It would be interesting to make a comparative study in the future to investigate whether demographic variables (e.g., age, gender, and education) could make an impact on the textual assessment and hybrid model.

Last but not least, since the data used in this study was collected via an online platform, special caution needs to be taken as far as the potential risk of fake information. We had invited at least two psychiatrists to check each self-narrative entry to ensure the input was reasonable and authentic and could be used in this study. However, how to validate the internet data before entering data processing would be an important topic. For instance, He et al. (2014a) introduced an approach to detect potential fake information on social media (i.e., Facebook) data collection via statistical models on person and item fit.

Prevalence of a condition is an important indicator when reporting the performance metrics of a screening method. Whereas sensitivity and specificity are independent of the prevalence of the disorder in the population, positive and negative predictive power are sensitive to population prevalence (Brewin, 2005). In our previous study (He et al., 2014b), we reported the possible prevalence as ranging from 5 to 50% and noticed that there was little difference in the accuracy of screening for PTSD using the PSM model when the range of prevalence was so large. It was also noticed that when the prevalence of PTSD in the sample was increased, the PPV increased as well. It meant that the confidence of correctly identifying PTSD also increased. In the current study, we note that both specificity and PPV increased when we used the hybrid model.

In summary, the current study presented a new trial in developing a hybrid model to combine textual assessment of patients’ self-narratives and itemized questionnaire in detecting mental illness. Its aim was to reduce the respondents’ burden and clinicians’ workload. Adding textual prior information, detection accuracy could be enhanced and test length could be shortened. The results demonstrated that the combination of a textual assessment and an IRT-based questionnaire is a promising approach to increase cost-effectiveness in PTSD diagnosis and is expected to be applicable in a broader scope of both (online) screening and psychiatric diagnosis as well as other psychological and educational assessments in the future. Further, with the rapid development of computer-based assessments, more data could be captured during the assessment process. The use of timing data as well as action sequences, keystrokes (e.g., type in and delete), and other process-related information hold promise for contributions to the advancement of screening methods in future research.

## Ethics Statement

This study was carried out in accordance with the recommendations of Code of Ethics for Research in the Social and Behavioural Sciences Involving Human Participants used as the guidelines by the Faculty of Behavioural, Management and Social Sciences (BMS) Ethics Committee, University of Twente with written informed consent from all subjects. All subjects gave written informed consent in accordance with the Declaration of Helsinki. The protocol was approved by the BMS Ethics Committee, University of Twente.

## Author Contributions

QH contributed to the development of the methodological framework and the model estimation procedures, conduction of the data analysis, and the drafting and revision of the manuscript. BV contributed to providing suggestions on the methodological framework and the model estimation procedures, and the reviewing and revision of the manuscript. CG contributed to providing suggestions on the methodological framework, and the reviewing of the manuscript. SB contributed to providing suggestions on the model estimation procedures and conduction of the data analysis, and the reviewing of the manuscript.

## Conflict of Interest

The authors declare that the research was conducted in the absence of any commercial or financial relationships that could be construed as a potential conflict of interest.

## Footnotes

1 The current study used the standard “English Stop Word List” (127 words) in Python Natural Language Toolkit (NLTK, Perkins, 2010) to deduct the non-informative words.

2 The stemming algorithm is used to normalize lexical forms of words, which may generate stems without an authentic word meaning, such as “*nightmar.*”

3 A body of texts is usually called a text corpus.

4 In He et al. (2014b), the IRT parameters were calculated by the marginal maximum likelihood method with the assumption that ability was in a standard normal distribution. The original ability scale was therefore also in a standard normal distribution. In other words, after fixing the IRT parameters, the resulting ability scores are on a standard normally distributed scale. Therefore we can normalize the text score on the same scale.

5 Only people who had mental health problems or were screened as positively high potential into mental problems in the round 1 were included as a target sample of PTSD in the NCS-R.

## References

[B1] Alvarez-Conrad J. Zoellner L. A. Foa E. B. (2001). Linguistic predictors of trauma pathology and physical health. *Appl. Cogn. Psychol.* 15 159–170.

[B2] American Psychiatric Association (2000). *Diagnostic and Statistical Manual of Mental Disorders: DSM-IV.* Washington, DC: Author.

[B3] Bock R. D. Aitkin M. (1981). Marginal maximum likelihood estimation of item parameters: an application of an EM-algorithm. *Psychometrika* 46 443–459. 10.1007/bf02293801

[B4] Brandon P. R. (2002). Two versions of the contrasting-groups standard-setting method: a review. *Measur. Eval. Counsel. Dev.* 35 167–181. 10.1080/07481756.2002.12069061

[B5] Brewin C. R. (2005). Systematic review of screening instruments for adults at risk of PTSD. *J. Trauma. Stress* 18 53–62. 10.1002/jts.20007 16281196

[B6] Brewin C. R. Andrews B. Valentine J. D. (2000). Meta-analysis of risk factors for posttraumatic stress disorder in trauma-exposed adults. *J. Consult. Clin. Psychol.* 68 748–766. 10.1037/0022-006x.68.5.748 11068961

[B7] Cizek G. J. Bunch M. B. (2007). *Standard Setting: A Guide to Establishing and Evaluating Performance Standards on Tests.* Thousand Oaks, CA: Sage.

[B8] Connor K. Davidson J. (2001). Sprint: a brief global assessment of post-traumatic stress disorder. *Int. Clin. Psychopharmacol.* 16 279–284. 10.1097/00004850-200109000-00005 11552771

[B9] Diamond E. L. Lilienfeld A. M. (1962). Effects of errors in classification and diagnosis in various types of epidemiological studies. *Am. J. Public Health* 52 1137–1144. 10.2105/ajph.52.7.1137 13886107 PMC1522932

[B10] Elhai J. D. de Francisco Carvalho L. Miguel F. K. Palmieri P. A. Primi R. Frueh B. C. (2011). Testing whether postraumatic stress disorder and major depressive disorder are similar or unique constructs. *J. Anxiety Disord.* 25 404–410. 10.1016/j.janxdis.2010.11.003 21129914

[B11] Franklin C. L. Thompson K. E. (2005). Response style and posttraumatic stress disorder (PTSD): a review. *J. Trauma Dissociation* 6 105–123. 10.1300/j229v06n03_05 16172084

[B12] Glas C. A. W. (1998). Detection of differential item functioning using lagrange multiplier tests. *Stat. Sin.* 8 647–667.

[B13] Glas C. A. W. (1999). Modification indices for the 2-PL and the nominal response model. *Psychometrika* 64 273–294. 10.1007/BF02294296

[B14] Gottschalk L. A. Gleser G. C. (1969). *The Measurement of Psychological States Through the Content Analysis of Verbal Behavior.* Berkeley, CA: University of California Press.

[B15] Gray M. J. Elhai J. D. Owen J. R. Monroe R. (2009). Psychometric properties of the trauma assessment for adults. *Depress. Anxiety* 26 190–195. 10.1002/da.20535 19031486

[B16] Gray M. J. Litz B. T. Hsu J. L. Lombardo T. W. (2004). Psychometric properties of the life events checklist. *Assessment* 11 330–341. 10.1177/1073191104269954 15486169

[B17] He Q. (2013). *Text Mining and IRT for Psychiatric and Psychological Assessment.* Enschede: Universiteit Twente, 10.3990/1.9789036500562

[B18] He Q. Glas C. A. W. Kosinski M. Stillwell D. J. Veldkamp B. P. (2014a). Predicting self-monitoring skills using textual posts on Facebook. *Comput. Hum. Behav.* 33 69–78. 10.1016/j.chb.2013.12.026

[B19] He Q. Glas C. A. W. Veldkamp B. P. (2014b). Assessing the impact of differential symptom endorsement on posttraumatic stress disorder (PTSD) diagnosis. *Int. J. Methods Psychiatr. Res.* 23 131–141. 10.1002/mpr.1417 24436035 PMC6878299

[B20] He Q. Veldkamp B. P. (2012). “Classifying unstructured textual data using the product score model: an alternative text mining algorithm,” in *Psychometrics in Practice at RCEC*, eds Eggen T. J. H. M. Veldkamp B. P., (Enschede: RCEC), 47–62.

[B21] He Q. Veldkamp B. P. de Vries T. (2012). Screening for posttraumatic stress disorder using verbal features in self narratives: a text mining approach. *Psychiatry Res.* 198 441–447. 10.1016/j.psychres.2012.01.032 22464046

[B22] He Q. Veldkamp B. P. Glas C. A. W. de Vries T. (2017). Automated assessment of patients’ self-narratives for posttraumatic stress disorder screening using natural language processing and text mining. *Assessment* 24 157–172. 10.1177/1073191115602551 26358713

[B23] Jurafsky D. Martin J. H. (2009). *Speech and Language Processing: An Introduction to Natural Language Processing, Computational Linguistics, and Speech Recognition.* Upper Saddle River, NJ: Pearson Prentice Hall.

[B24] Kessler R. C. Berglund P. Chiu W. T. Demler O. Heeringa S. Hiripi E. (2004). The US national comorbidity survey replication (NCS-R) design and field procedures. *Int. J. Methods Psychiatr. Res.* 13 69–92. 10.1002/mpr.167 15297905 PMC6878537

[B25] Kubany E. S. Leisen M. B. Kaplan A. S. Watson S. B. Haynes S. N. Owens J. A. (2000). Development and preliminary validation of a brief broad-spectrum measure of trauma exposure: the traumatic life events questionnaire. *Psychol. Assess.* 12 210–224. 10.1037//1040-3590.12.2.210 10887767

[B26] Lancaster C. L. Teeters J. B. Gros D. F. Back S. E. (2016). Posttraumatic stress disorder: overview of evidence-based assessment and treatment. *J. Clin. Med.* 5:105. 10.3390/jcm5110105 27879650 PMC5126802

[B27] Lang A. J. Stein M. B. (2005). An abbreviated PTSD checklist for use as a screening instrument in primary care. *Behav. Res. Ther.* 43 585–594. 10.1016/j.brat.2004.04.005 15865914

[B28] Laplace P. S. (1995). *Pierre-Simon Laplace Philosophical Essay on Probabilities*, Vol. 13 New York, NY: Springer.

[B29] Lord F. M. (1980). *Applications of Item Response Theory to Practical Testing Problems.* Hillsdale, CA: Erlbaum.

[B30] Lunn D. J. Thomas A. Best N. Spiegelhalter D. (2000). WinBUGS: a bayesian modeling framework: concepts, structure, and extensibility. *Stat. Comput.* 10 325–337.

[B31] Manning C. D. Schütze H. (1999). *Foundations of Statistical Natural Language Processing.* Cambridge, MA: MIT Press.

[B32] Matteucci M. Veldkamp B. P. (2013). On the use of MCMC computerized adaptive testing with empirical prior information to improve efficiency. *Stat. Methods Appl.* 22 243–267. 10.1007/s10260-012-0216-1

[B33] Mislevy R. J. (1986). Bayes modal estimation in item response models. *Psychometrika* 51 177–195. 10.1007/bf02293979

[B34] Naglieri J. A. Drasgow F. Schmit M. Handler L. Prifitera A. Margolis A. (2004). Psychological testing on the internet. *Am. Psychol.* 59 150–162. 15222858 10.1037/0003-066X.59.3.150

[B35] Orsillo S. M. Batten S. V. Plumb J. C. Luterek J. A. Roessner B. M. (2004). An experimental study of emotional responding in women with posttraumatic stress disorder related to interpersonal violence. *J. Trauma. Stress* 17 241–248. 10.1023/b:jots.0000029267.61240.94 15253096

[B36] Pennebaker J. W. (2001). Dealing with a traumatic experience immediately after it occurs. *Adv. Mind Body Med.* 17 160–162. 11572841 10.1054/ambm.2000.0307

[B37] Pennebaker J. W. Francis M. E. Booth R. J. (2001). *Linguistic Inquiry and Word Count.* Mahwah, NJ: Erlbaum.

[B38] Pennebaker J. W. Mehl M. R. Niederhoffer K. G. (2003). Psychological aspects of natural language. use: our words, our selves. *Annu. Rev. Psychol.* 54 547–577. 10.1146/annurev.psych.54.101601.145041 12185209

[B39] Perkins J. (2010). *Python Text Processing with NLTK 2.0 Cookbook*. Birmingham: Packt Publishing Ltd.

[B40] Porter M. F. (1980). An algorithm for suffix stripping. *Program Autom. Library Inform. Syst.* 14 130–137. 10.1108/eb046814

[B41] Prins A. Ouimette P. (2004). The primary care PTSD screen (PC-PTSD): development and operating characteristics. *Primary Care Psychiatry* 9 9–14. 10.1016/j.jad.2019.05.021 31158781

[B42] Rasch G. (1960). *Probabilistic Models for Some Intelligence and Attainment Tests.* Copenhagen: Danish Institute for Educational Research.

[B43] Rosenberg S. D. Tucker G. J. (1979). Verbal-behavior and schizophrenia—semantic dimension. *Arch. Gen. Psychiatry* 36 1331–1337. 496553 10.1001/archpsyc.1979.01780120061008

[B44] Schnurr P. P. Spiro A. Vielhauer M. J. Findler M. N. Hamblen J. L. (2002). Trauma in the lives of older men: findings from the normative aging study. *J. Clin. Geropsychol.* 8 175–187.

[B45] Shrout P. E. Skodol A. E. Dohrenwend B. P. (1986). “A two-stage approach for case identification and diagnosis, first stage instruments,” in *Mental Disorders in Community: Progress and Challenge*, eds Barrett J. E. Rose R. M., (New York, NY: Guilford), 286–303.

[B46] Shrout P. E. Yager T. J. (1989). Reliability and validity of screening scales: effects of reducing scale length. *J. Clin. Epidemiol.* 42 69–78. 10.1016/0895-4356(89)90027-9 2913189

[B47] Smyth J. M. (1998). Written emotional expression: effect sizes, outcome types and moderating variables. *J. Consult. Clin. Psychol.* 66 174–184. 10.1037/0022-006x.66.1.174 9489272

[B48] Thomas M. L. (2011). The value of item response theory in clinical assessment: a review. *Assessment* 18 291–307. 10.1177/1073191110374797 20644081

[B49] Todorov G. I. Mayilvahanan K. Cain C. K. Cunha C. (2018). *Screening Word Usage in People Affected by PTSD: An Unbiased, Cost Effective, and Novel Screening Method*? *PsyArXiv* (Preprints). Available at: https://psyarxiv.com/y68fx/ (accessed October 10, 2019).

[B50] van den Berg S. M. Paap M. C. S. Derks E. M. Genetic Risk and Outcome of Psychosis (Group) investigators (2013). Using multidimensional modeling to combine self-report symptoms with clinical judgment of schizotypy. *Psychiatry Res.* 206 75–80. 10.1016/j.psychres.2012.09.015 23021911

[B51] van der Linden W. J. Glas C. A. (eds). (2000). *Computerized Adaptive Testing: Theory and Practice*. Dordrecht: Kluwer Academic.

[B52] van Groen M. M. ten Klooster P. M. Taal E. van de Laar M. A. F. J. Glas C. A. W. (2010). Application of the health assessment questionnaire disability index to various rheumatic diseases. *Qual. Life Res.* 19 1255–1263. 10.1007/s11136-010-9690-9 20559736 PMC2963741

[B53] Weisscher N. Glas C. A. W. Vermeulen M. de Haan R. J. (2010). The use of an item response theory-based disability item bank across diseases: accounting for differential item functioning. *J. Clin. Epidemiol.* 63 543–549. 10.1016/j.jclinepi.2009.07.016 19880281

[B54] Wohlfarth T. van den Brink W. Winkel F. W. ter Smitten M. (2003). Screening for posttraumatic stress disorder: an evaluation of two self-report scales among crime victims. *Psychol. Assess.* 15 101–109. 10.1037/1040-3590.15.1.101 12674729

[B55] Wong E. Ungvari G. S. Leung S. K. Tang W. K. (2007). Rating catatonia in patients with chronic schizophrenia: rasch analysis of the bush-francis catatonia rating scale. *Int. J. Methods Psychiatr. Res.* 16 161–170. 10.1002/mpr.224 17849434 PMC6878392

[B56] Zwinderman A. H. (1991). A generalized Rasch model for manifest predictors. *Psychometrika* 56 589–600. 10.1007/bf02294492

